# Integrating Helicase-Dependent and Rolling Circle Amplification in a Single Tube for Colorimetric Detection of *Staphylococcus aureus*

**DOI:** 10.3390/diagnostics16081131

**Published:** 2026-04-09

**Authors:** Polina Chirkova, Dmitry Gryadunov, Alexander Chudinov, Sergey Lapa

**Affiliations:** Engelhardt Institute of Molecular Biology (EIMB), Russian Academy of Sciences, 119991 Moscow, Russia

**Keywords:** helicase-dependent amplification, rolling circle amplification, colorimetric detection, *Staphylococcus aureus*

## Abstract

**Background/Objectives**: Rapid, equipment-free molecular detection of bacterial pathogens at the point of care (POC) remains a critical challenge. *Staphylococcus aureus* is a leading cause of severe infections, necessitating simple and sensitive diagnostic tools. **Methods**: We developed an integrated assay combining helicase-dependent amplification (HDA) and rolling circle amplification (RCA) in a sequential ‘one-pot’ format. Asymmetric HDA generates short, single-stranded amplicons from *S. aureus* DNA, enabling specific padlock probe ligation and subsequent exponential RCA. For equipment-free visual detection, biotin-labeled nucleotides are incorporated during RCA, and products are captured on a silica membrane and detected using a streptavidin-HRP conjugate with 3,3′,5,5′-tetramethylbenzidine substrate, producing an unambiguous blue color. **Results**: The assay detected as few as 10^1^ genome copies of *S. aureus* per reaction. Evaluation against a panel of nine non-target respiratory pathogens and human genomic DNA demonstrated 100% specificity, with no cross-reactivity. The entire procedure is performed isothermally at 65 °C in a single tube with a total assay time of approximately 90 min. **Conclusions**: This ‘one-pot’ HDA-RCA colorimetric assay combines high sensitivity and specificity for *S. aureus* in a user-friendly, almost equipment-free format. Its simplicity and robust visual readout make it a promising tool for POC diagnostics in resource-limited settings, enabling rapid clinical decisions without specialized instrumentation.

## 1. Introduction

Rapid and accurate detection of bacterial pathogens in point-of-care (POC) or resource-limited settings remains one of the key challenges in global health [1]. Traditional diagnostics rely heavily on labor-intensive microorganism cultivation (24–48 h), whereas the molecular gold standard, quantitative polymerase chain reaction (qPCR), requires sophisticated thermocyclers and trained personnel, limiting its use to centralized laboratories [2,3]. *Staphylococcus aureus* is a leading cause of severe hospital-acquired and community-acquired infections, accounting for approximately 35–48% of culture-confirmed surgical site infections. It is also a major pathogen in periprosthetic joint infections, with treatment failure rates reaching up to 46% [4,5]. Consequently, there is an urgent need for diagnostic platforms that combine high analytical sensitivity and specificity with ease of use, rapid time-to-result, and minimal equipment requirements [6].

Isothermal nucleic acid amplification techniques (INAATs) have emerged as a powerful alternative to PCR for point-of-care applications due to their ability to operate at a constant temperature [7]. Among these methods, rolling circle amplification (RCA) is distinguished by its exceptional specificity, conferred by a precise target-dependent ligation step, and its inherent capacity for signal amplification through the generation of long amplicons containing tandem repeats from a circular DNA template [8]. Although RCA has been successfully applied to detect various targets, including single nucleotide polymorphisms and short viral RNA [9,10,11], its application to long single-stranded RNA and double-stranded DNA is hampered by steric inaccessibility of the target for effective padlock probe ligation, necessitating a sample preparation step [12,13].

A common strategy to overcome this limitation is to preamplify the genomic target, generating shorter, more accessible fragments. Although PCR-based preamplification is effective, it reintroduces the requirement for thermal cycling, which complicates assay integration and undermines the goal of a simple isothermal workflow [1]. Helicase-dependent amplification (HDA) offers an acceptable alternative as a truly isothermal method, employing DNA helicase to unwind the DNA template and enable primer annealing and strand-displacement synthesis at a single temperature [14,15].

We previously demonstrated the feasibility of a combined HDA-RCA approach that significantly improves analytical sensitivity for the detection of full-length bacterial DNA by generating short, predominantly single-stranded amplicons that are optimal for subsequent padlock probe ligation and RCA [16]. However, our proof-of-concept study relied on gel electrophoresis for end-point detection, a method poorly suited to point-of-care settings. Although HDA alone can provide adequate amplification for certain detection formats, its sensitivity may be insufficient for direct visual readout without further signal enhancement [17].

The ultimate goal of POC molecular diagnostics is to develop a fully integrated assay in which all reaction steps occur sequentially in a single tube, resulting in simple, instrument-free, and preferably colorimetric detection. Recent advances have demonstrated the successful integration of isothermal amplification with various colorimetric detection strategies, each offering distinct advantages and limitations. Broadly, these strategies can be classified into four main categories.

Metal-ion indicators, such as hydroxynaphthol blue (HNB), enable simple, one-step detection by changing color in response to decreasing Mg^2+^ concentrations resulting from pyrophosphate formation during amplification. However, they may suffer from limited sensitivity and subjective color interpretation [18,19].

pH indicators, such as phenol red, provide a low-cost option by detecting protons released during DNA synthesis, but their performance can be influenced by buffer capacity and sample composition, potentially leading to false positives or negatives [20].

Dye-based and probe-based methods, including intercalating dyes (e.g., malachite green, leuco crystal violet) and functionalized gold nanoparticles (AuNPs), offer improved specificity through sequence-dependent detection. However, intercalating dyes may require specialized equipment for optimal visualization, while AuNP-based assays can be sensitive to reaction conditions and require careful optimization [19,21,22].

Enzyme-based systems, most commonly employing horseradish peroxidase (HRP) in combination with chromogenic substrates such as 3,3′,5,5′-tetramethylbenzidine (TMB), represent an alternative approach where signal generation is coupled to the amplification product through labeled nucleotides (e.g., biotin-dUTP) and subsequent affinity capture [23]. Unlike methods that rely on amplification by-products, enzyme-based detection directly targets the amplified DNA, potentially offering enhanced specificity and eliminating the ambiguity associated with subtle color changes in pH or metal indicators [19]. Furthermore, the HRP-TMB system produces an intense, stable blue color that is easily discernible by the naked eye, making it particularly attractive for equipment-free applications.

This approach could be ideally suited for integration with isothermal amplification methods such as the proposed HDA-RCA assay, where biotinylated nucleotides can be incorporated during the amplification stage, enabling subsequent solid-phase capture and washing to isolate the signal from complex reaction mixtures. Thus, HRP-based colorimetric detection presents a promising strategy for developing fully integrated, equipment-free molecular assays for point-of-care applications.

In this study, we report the development and validation of a novel ‘one-pot’ HDA-RCA colorimetric assay for highly sensitive, equipment-free detection of *S aureus*. Building on our previous work [16], we developed a sequential single-tube protocol that combines asymmetric HDA for initial target amplification, padlock probe ligation, and exponential RCA for signal amplification. For visual detection, we incorporated a colorimetric readout based on the HRP-TMB system, in which biotin-labeled RCA products are captured on a silica membrane and detected via a streptavidin-HRP conjugate, producing a distinct blue color in the presence of the target. We evaluated the analytical sensitivity of the assay, its specificity against a panel of clinically relevant bacterial species, and its performance using DNA from both reference strains and clinical isolates.

## 2. Materials and Methods

### 2.1. Bacterial Strains and DNA Isolation

Genomic DNA of *S. aureus* strains and other bacterial species studied was isolated at the State Research Center for Applied Microbiology and Biotechnology (Obolensk, Russia) [24]. DNA was extracted from reference strains and clinical isolates using the PREP-MB-RAPID II DNA/RNA Extraction Kit (IVD approval for the Russian Federation, cat. # P-124-P/9EU, DNA-Technology, LLC, Moscow, Russia). The concentration of the extracted DNA was measured using a NanoDrop 2000 spectrophotometer (Thermo Fisher Scientific, Wilmington, DE, USA). For specificity assessment, genomic DNA from a panel of clinically relevant bacterial species and human genomic DNA (from a reference set) were used (Table 1). The species identity of *S. aureus* isolates, as well as the presence or absence of methicillin resistance determinants, was confirmed using the reference “AmpliSens^®^ MRSA-screen-titer-FL” kit for the detection and quantification of MRSA (Central Research Institute of Epidemiology, Moscow, Russia), according to the manufacturer’s instructions.

### 2.2. Oligonucleotides and Primers

The *ebpS* gene (elastin-binding protein) was selected as the target because it is present in the vast majority of *S. aureus* clinical isolates, making it a reliable marker for species identification [25].

Primers were designed using the Integrated DNA Technologies web resource (www.idtdna.com, accessed on 19 September 2025), and specificity analysis was performed using the BLASTn v2.16.0 algorithm (National Institutes of Health, Bethesda, MD, USA).

Two primers were developed for HDA: forward 5′-TTCTTTATCTCTGTCATGATTGTCATGTTC-3ʹ and reverse 5′-CAGCAAGTAAAAGTGCTTCTGCCGCTTC-3ʹ. These primers target the highly species-specific region of the *ebpS* gene encoding the staphylococcal elastin-binding protein. A 90-mer padlock probe (circular RCA template) was designed and synthesized with the following sequence phosphorylated at the 5′ end: 5′-Ph-TTAGAGGCATGTGGTTATGCTAGCAACAGATAGACAAGATGGAATGCAGATGACGATAGACGATACCTGCCATCGTTTTGGCTTGCATTA-3′, where Ph represents a phosphate group. For RCA, the primer 5′-GCATTCCATCTTGTCTATCTGTTGCTAGC-3′, specific to the non-coding region of the circularized probe, was used.

Solid-phase oligonucleotide synthesis was performed using an ABI 394 automatic DNA/RNA synthesizer (Applied Biosystems, Foster City, CA, USA) according to the standard protocol. Purification was carried out on a BDS Hypersil C18 column (Thermo Scientific, Carlsbad, CA, USA). For the synthesis of relatively long padlock probes, a modified protocol was employed, with the condensation step extended to 35 s to increase the yield of the full-length oligonucleotide. Reduced column loading was used for purification of such long oligonucleotides.

### 2.3. ‘One-Pot’ Sequential HDA-RCA Protocol

Reaction conditions, including primer ratios (10:1), enzyme concentrations, and incubation times, were optimized in preliminary experiments during the development of the method to achieve the maximal signal-to-noise ratio while maintaining specificity. The entire assay was performed in a single 0.2 mL PCR tube in a final volume of 60 μL, with all incubation steps carried out at 65 °C in a conventional heating block (Termo 24-15, Biocom, Moscow, Russia) or a thermal cycler (e.g., Gentier 96E, Tianlong, Xi’an, China; or GeneExplorer GE-48DG, Bioer, Hangzhou, China). The reaction was initiated with 20 μL of HDA mixture containing 2.1 μL of IsoAmp Enzyme Mix (New England Biolabs, Ipswich, MA, USA) in buffer (4 mM Tris-HCl, 2 mM (NH_4_)_2_SO_4_, 10 mM KCl, 0.4 mM MgSO_4_, 0.02% Tween 20), forward and reverse primers at 0.2 μM and 0.02 μM, respectively, to generate predominantly single-stranded products, and template DNA ranging from 10^1^ to 10^5^ genome copies. After 30 min of incubation, the tube was briefly opened for the addition of 20 μL of ligation mixture containing 0.2 μM of the 90 nt padlock probe, 40 units of 9° N DNA ligase (New England Biolabs, Ipswich, MA, USA) in buffer (4 mM Tris-HCl, 120 μM ATP, 0.5 mM DTT_2_, 0.02% Tween 20), followed by another 30 min of incubation for probe hybridization and circularization. Subsequently, 20 μL of RCA mixture containing 200 μM dNTPs, 1.6 units of BST 3.0 DNA polymerase (New England Biolabs, Ipswich, MA, USA) in buffer (4 mM Tris-HCl, 3 mM (NH_4_)_2_SO_4_, 30 mM KCl, 0.4 mM MgSO_4_, 0.02% Tween 20), 0.5 μM RCA primer, and 6 μM biotin-11-dUTP (Lumiprobe, Moscow, Russia) were added. The tube was then incubated for a final 30 min at 65 °C.

### 2.4. Colorimetric Detection

**Figure 1:**
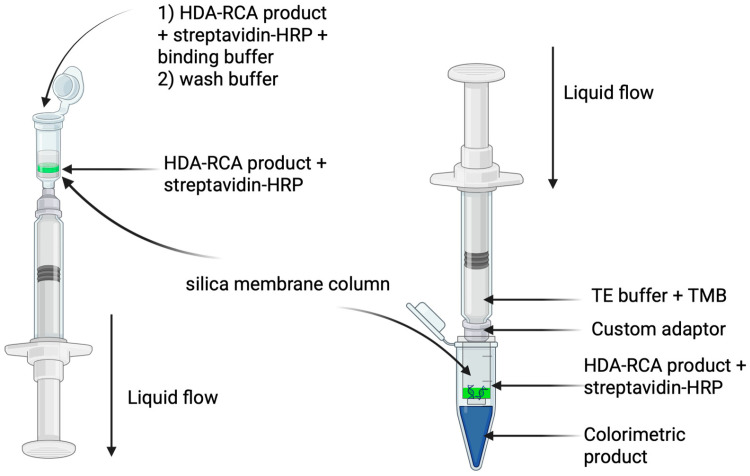
Schematic illustration of the two-step colorimetric detection procedure using a syringe-driven silica membrane column. (**Left**) Loading and washing: The biotin-labeled RCA product is mixed with streptavidin-HRP conjugate and binding buffer, then drawn through the column by pulling the syringe plunger. Unbound components are removed by two consecutive washes with wash buffer. (**Right**) Detection: TMB substrate is pushed through the column, where it reacts with the immobilized HRP, producing a blue color in the eluate. Created in BioRender. Chirkova, P. (2026) https://BioRender.com/o8lzcfz (accessed on 13 March 2026).

The procedures required for visual colorimetric determination were performed at room temperature. A schematic representation of the custom column system is shown in Figure 1. The system consisted of a standard silica-gel membrane column (Dia-M, Moscow, Russia) modified for syringe-driven flow. The protocol involved two sequential configurations. First (Figure 1, left), a 10 mL disposable syringe was securely attached to the bottom of the column via a Luer-Lock adapter. In this setup, 100 μL of the HDA-RCA product was mixed with 40 μL of streptavidin-HRP conjugate solution (Thermo Fisher Scientific, Carlsbad, CA, USA) diluted to 0.5 μg/mL in 1× PBS (pH 7.2) and 400 μL of binding buffer (Dia-M, Moscow, Russia). The mixture was applied to the top of the column. The syringe plunger was then slowly pulled down, creating a pressure differential that gently drew the mixture through the membrane and into the syringe, allowing the target to bind to the silica while simultaneously collecting the flow-through. To remove unbound components, two consecutive washes with 700 μL of wash buffer (consisting of 96% ethanol and 2× PBS (pH 7.2) mixed in a 50:50 ratio (*v*/*v*)) were performed by carefully applying the buffer to the top of the column and gently pulling it through the membrane using the same syringe after each application. Thus, a single syringe attached at the bottom was sufficient for all three loading and wash steps, efficiently collecting both the initial flow-through and subsequent wash fractions. For the detection step (Figure 1, right), the column was transferred to a fresh collection tube. A new syringe, pre-filled with 100 μL of elution solution (TE buffer, pH 8.0) containing 40 μL of TMB substrate (Niopic, Dolgoprudny, Russia), was attached to the top of the column. The plunger was then slowly depressed, pushing the substrate through the membrane. The pulling and pushing steps were performed manually at a slow, steady rate (approximately 5–10 s per full stroke) to ensure consistent flow. Preliminary experiments showed that variation within this range did not affect the final signal. As the TMB interacted with the immobilized peroxidase, a color change was observed in the eluate, providing a visual signal detectable after 5 min of incubation.

### 2.5. Limit of Detection Estimation

The limit of detection (LoD) was defined as the lowest DNA concentration yielding a positive colorimetric result in at least 95% of replicates. Probit regression analysis was performed using IBM SPSS Statistics for Windows, Version 26.0 (IBM Corp, Armonk, NY, USA) on the binary outcomes (positive/negative) obtained from three independent replicates at each concentration (10^2^, 10^3^, 10^4^, 10^5^ copies/reaction), nine independent replicates at 10^1^ and 10^0^ copies/reaction, and the negative control. The consistency of the binary (positive/negative) results across replicates was recorded. The LoD with 95% confidence interval was calculated from the fitted probit model.

## 3. Results

### 3.1. Development of a Sequential ‘One-Pot’ HDA-RCA Protocol

**Figure 2:**
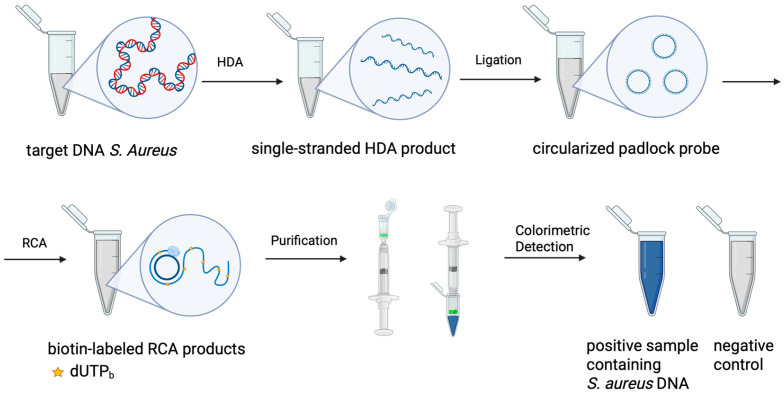
Schematic representation of the ‘one-pot’ HDA-RCA assay. The protocol includes three sequential steps at 65 °C: (1) asymmetric HDA preamplification; (2) padlock probe ligation; and (3) exponential RCA with biotin-dUTP incorporation (dUTP_b_). The biotin-labeled amplification products are then visualized using a streptavidin-HRP-TMB colorimetric system, producing a blue signal for positive samples. Created in BioRender. Chirkova, P. (2026) https://BioRender.com/wtewdnh (accessed on 13 March 2026).

We developed a sequential, single-tube protocol that temporally separates the three enzymatic stages while preserving the ‘one-pot’ format (Figure 2). The optimized workflow consists of (i) a 30 min asymmetric HDA preamplification step using only forward and reverse primers (at a 10:1 ratio to generate predominantly single-stranded products); (ii) a brief opening of the tube for the addition of the ligation mixture (padlock probe and 9N DNA ligase), followed by a 30 min incubation for probe hybridization and circularization; and (iii) a final addition of the RCA mixture (containing BST 3.0 polymerase, primers, dNTPs, and biotin-11-dUTP) with a subsequent 30 min incubation (DNA amplification). All steps were performed isothermally at 65 °C. This temporal separation successfully minimized interference between reaction components, enabling robust and reproducible amplification.

To enable equipment-free visual readout, biotin-labeled nucleotides (biotin-11-dUTP) were incorporated into the RCA products during the amplification step. After completion of the ‘one-pot’ reaction, the biotinylated RCA products were captured on a silica-gel membrane column, washed to remove unbound components, and detected using a streptavidin-HRP conjugate with TMB substrate (Figure 1).

**Figure 3:**
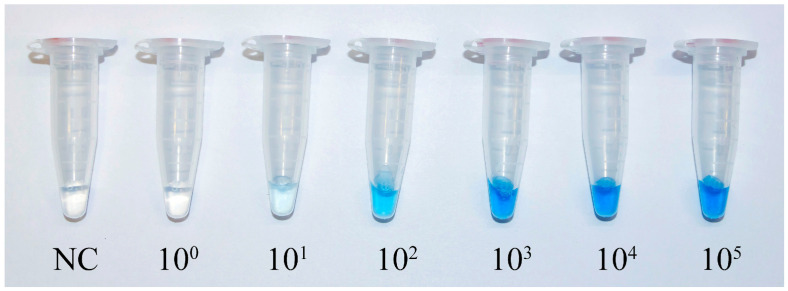
Colorimetric detection of *S. aureus* DNA using the ‘one-pot’ HDA-RCA assay. Serial dilutions of genomic DNA (10^0^–10^5^ copies/reaction) produced blue color proportional to target concentration, with a detection limit of 10^1^ copies. The negative control (NC) showed no color development. Results are representative of three independent experiments.

As shown in Figure 3, samples containing *S. aureus* DNA developed an intense blue color within five minutes of substrate addition. The negative control sample remained completely colorless, confirming that (i) in the absence of target, no biotin-labeled RCA products are generated; and (ii) non-specific adsorption of the streptavidin-HRP conjugate to the column matrix is effectively eliminated by the optimized washing conditions. This colorimetric readout provides an unambiguous, naked-eye detectable signal without the need for any specialized instrumentation.

### 3.2. Analytical Sensitivity of the Integrated Assay

The analytical sensitivity of the ‘one-pot’ HDA-RCA assay was evaluated using serial ten-fold dilutions of purified *S. aureus* genomic DNA (ATCC 25923 and 224/228 MRSA strains), ranging from 10^0^ to 10^5^ genome copies per reaction. For concentrations of 10^2^ copies and above, each was tested in three replicates; for 10^1^ copies and below, each was tested in nine replicates (three independent experiments with three technical replicates each). Probit regression analysis performed on the combined data yielded an LoD (95% probability of detection) of 9.3 genome copies per reaction (95% CI: 4.7–28.1 copies). At the nominal concentration of 10^1^ copies, the detection rate was 100% (9/9 replicates), confirming that the assay reliably detects as few as 10^1^ copies. No positive signals were observed at 10^0^ copies or in the negative control (0/9 replicates each).

The reproducibility of the assay was assessed across all tested concentrations. For 10^2^ copies and above, all three replicates yielded identical positive results. For 10^1^ copies, all nine replicates were positive. For 10^0^ copies and the negative control, all replicates (nine each) were negative. Therefore, 100% concordance was observed at every concentration, demonstrating robust reproducibility of the qualitative readout. Following the complete sequential reaction and colorimetric detection, a distinct blue color was observed for all samples containing *S. aureus* DNA, while the NC remained completely colorless (Figure 3). The assay consistently detected as few as 10^1^ genome copies per reaction, although this lowest concentration produced a faint blue coloration. Samples containing 10^2^ copies yielded a clearly distinguishable blue color, while concentrations of 10^3^ copies and above resulted in intense blue staining. Notably, the color intensity reached saturation at 10^3^ copies, with samples containing 10^4^ and 10^5^ copies showing virtually indistinguishable signals. This saturation effect is likely attributable to the combined amplification power of the sequential HDA and RCA steps, which efficiently amplify even moderately low target copies to levels saturating the colorimetric detection system.

### 3.3. Specificity of the ‘One-Pot’ HDA-RCA Assay

The specificity of the assay was evaluated against a panel of nine non-target bacterial species associated with respiratory infections, as well as human genomic DNA (Table 1). The panel included representatives of the genera *Streptococcus* (*S. pneumoniae*), *Legionella* (*L. pneumophila*), *Haemophilus* (*H. influenzae*), *Neisseria* (*N. lactamica*, *N. sicca*, *N. meningitidis*), *Pseudomonas* (*P. aeruginosa*), *Klebsiella* (*K. pneumoniae*), and *Mycobacterium* (*M. tuberculosis*). Additionally, DNA from eight different *S. aureus* strains (including deposited strains and clinical isolates, several of which were methicillin-resistant (MRSA)) was tested as positive controls.

No colorimetric signal was observed for any of the non-target bacterial species or for human DNA, indicating the absence of non-specific amplification or probe hybridization under the optimized reaction conditions. In contrast, all ten *S. aureus* samples produced a distinct blue color. No significant difference in detection limit (10^1^ copies) or signal intensity was observed between reference strains and clinical isolates. All ten *S. aureus* samples (five reference and five clinical) gave positive signals within the same time frame. These results demonstrate 100% specificity for the developed assay, with no cross-reactivity detected against a broad panel of clinically relevant respiratory pathogens or host DNA.

## 4. Discussion

The successful integration of HDA and RCA into a single-tube format addresses a key challenge in point-of-care molecular diagnostics: combining high analytical sensitivity with operational simplicity. Our sequential ‘one-pot’ approach overcomes the steric hindrance that typically limits direct RCA application to long double-stranded genomic DNA targets [12,13]. By first generating short, predominantly single-stranded amplicons via asymmetric HDA, we create optimal substrates for padlock probe hybridization and ligation, enabling the exponential signal amplification capability of RCA to be fully realized.

The temporal separation of the three enzymatic stages of HDA preamplification, probe ligation, and RCA, was critical to assay performance. Initial attempts to combine all components (HDA enzymes, primers, padlock probe, ligase, RCA polymerase, and dNTPs) in a single reaction mixture from the outset resulted in unstable amplification and frequent RCA failures. We hypothesize that this was due to competition between the HDA primers and the RCA primer for the target, as well as potential interference of the RCA enzyme mixture during the initial HDA phase. The sequential addition of components, while requiring a single tube-opening step, preserves the practical simplicity of the ‘one-pot’ format while ensuring robust and reproducible amplification. This design represents a pragmatic compromise between theoretical ideal (closed-tube) and practical necessity (compatibility of multiple enzymatic systems).

The achieved analytical sensitivity of 10^1^ genome copies per reaction places this assay among the most sensitive isothermal amplification methods reported for *S. aureus* detection [26]. This sensitivity is clinically relevant for direct testing of samples with moderate to low bacterial loads, such as blood or cerebrospinal fluid in early-stage infections [27]. The stage of peroxidase staining can be considered as an additional step of signal amplification. The observed signal saturation at ≥10^3^ copies reflects the high amplification efficiency of the coupled HDA-RCA system, which drives even low-abundance targets to saturating levels within the 90 min protocol. This saturation is advantageous for robust visual readout, as it minimizes well-to-well variability in color intensity at clinically relevant concentrations.

The 100% specificity demonstrated against a comprehensive panel of 9 non-target respiratory pathogens and human DNA is particularly noteworthy. This high specificity is conferred by two orthogonal molecular recognition events: (i) the HDA primers, which provide initial target selectivity, and (ii) the padlock probe ligation, which requires perfect sequence complementarity at the ligation junction. The absence of cross-reactivity with closely related species (e.g., other *Staphylococcus* species were not tested, but the panel included diverse Gram-positive and Gram-negative respiratory pathogens) suggests that the chosen target region is highly conserved within *S. aureus* yet sufficiently divergent from other clinically relevant bacteria. The inclusion of multiple MRSA further confirms that methicillin resistance does not affect assay performance.

The choice of HRP-TMB-based colorimetric detection offers several advantages over previously reported readout strategies for isothermal amplification. Unlike metal-ion indicators (e.g., HNB) or pH-sensitive dyes, which detect amplification by-products and can suffer from subjective color interpretation or buffer sensitivity [18,19,20], our approach directly detects the amplified DNA through incorporated biotin labels. This direct detection mechanism eliminates ambiguity: the blue color appears only when target-specific RCA products are generated. Furthermore, the solid-phase capture and washing steps effectively remove unincorporated biotin-dUTP and non-specifically bound conjugate, virtually eliminating false-positive signals. The intense blue color produced by the TMB substrate is easily discernible by the naked eye, making the assay almost equipment-free.

While the results are highly encouraging, several limitations should be acknowledged. First, the current protocol requires a single tube-opening step for addition of the ligation and RCA mixtures. Although this is a minor deviation from a true closed-tube system, it introduces a minimal risk of amplicon contamination. Future iterations could explore lyophilized reagent pellets or wax-barrier mechanisms to enable fully closed-tube operation. Second, the colorimetric detection system, while equipment-free, currently requires manual syringe-driven flow through the silica membrane. Integration into a simple, low-cost microfluidic device could further enhance user-friendliness and standardization. One more limitation of this study is that the specificity panel did not include other *Staphylococcus* species commonly found in clinical specimens, such as *S. epidermidis*, *S. saprophyticus*, or *S. haemolyticus*. Although the chosen *ebpS* target is reported to be highly specific for *S. aureus* [25], future validation should include these closely related species to further confirm the absence of cross-reactivity. Finally, while we have demonstrated excellent performance with purified genomic DNA, validation with clinical specimens (e.g., sputum, blood, and wound swabs) is essential to assess potential inhibition by complex biological matrices. Such studies are currently underway.

## 5. Conclusions

We have developed a ‘one-pot’ HDA-RCA colorimetric assay that enables equipment-free visual detection of *Staphylococcus aureus* with high sensitivity and specificity. The sequential integration of isothermal HDA preamplification with exponential RCA overcomes the steric limitations of direct RCA on genomic DNA, while the HRP-TMB colorimetric system provides an unambiguous naked-eye readout. The assay achieves a detection limit of 10^1^ genome copies per reaction and demonstrates 100% specificity against a broad panel of respiratory pathogens and human DNA. All steps were performed isothermally at 65 °C in a single tube, requiring only a simple heating block. This combination of high analytical performance, operational simplicity, and equipment-free detection makes the assay a promising tool for point-of-care diagnostics in resource-limited settings, where rapid and reliable *S. aureus* identification is critical for timely clinical decisions.

## Figures and Tables

**Table 1:** Strains and species used in this study.

ID	Species	Strain Designation	Source	Results of the ‘One-Pot’ Analysis
1	*Staphylococcus aureus*	ATCC 25923	deposited strain	+
2	*Staphylococcus aureus*	224\228 MRSA	deposited strain	+
3	*Staphylococcus aureus*	ATCC 700699 Mu 50	deposited strain	+
4	*Staphylococcus aureus*	ATCC BAA-1707 MRSA MW2	deposited strain	+
5	*Staphylococcus aureus*	ATCC 43300	deposited strain	+
6	*Staphylococcus aureus*	Orenburg_2014 #67	clinical isolate	+
7	*Staphylococcus aureus*	Ulyanovsk_2014 #2	clinical isolate	+
8	*Staphylococcus aureus*	Train_Abakan_2014 #3	clinical isolate	+
9	*Staphylococcus aureus*	Pskov_2014 #11526	clinical isolate	+
10	*Staphylococcus aureus*	Yakutia_2018 #Se49	clinical isolate	+
11	*Pseudomonas aeruginosa*	ATCC BAA-2470	deposited strain	−
12	*Haemophilus influenzae*	ATCC 9006, type A	deposited strain	−
13	*Streptococcus pneumoniae*	ATCC 6305	deposited strain	−
14	*Legionella pneumophila*	ATCC 33156	deposited strain	−
15	*Klebsiella pneumoniae*	KPB-944	deposited strain	−
16	*Neisseria lactamica*	ATCC 23970	deposited strain	−
17	*Neisseria sicca*	ATCC 9913	deposited strain	−
18	*Neisseria meningitidis*	ATCC 13077	deposited strain	−
19	*Mycobacterium tuberculosis*	H37Rv	deposited reference strain	−
20	*Homo sapiens*	-	human DNA sample from HLA-B27 real-time PCR genotyping kit (Cat. No. HLA-B27, DNA-Technology, LLC, Moscow, Russia	−

## Data Availability

The raw data supporting the conclusions of this article will be made available by the authors on request.

## Abbreviations

The following abbreviations are used in this manuscript:

HDA	Helicase-depended amplification
RCA	Rolling circle amplification
POC	Point-of-care
qPCR	Quantitative polymerase chain reaction
INAAT	Isothermal nucleic acid amplification techniques
HNB	Hydroxynaphthol blue
TMB	3,3′,5,5′-tetramethylbenzidine
HRP	Horseradish peroxidase
dNTPs	Deoxyribonucleotide triphosphates
